# Genetic analysis of members of the species *Oropouche virus* and identification of a novel M segment sequence

**DOI:** 10.1099/vir.0.000108

**Published:** 2015-07

**Authors:** Natasha L. Tilston-Lunel, Joseph Hughes, Gustavo Olszanski Acrani, Daisy E. A. da Silva, Raimunda S. S. Azevedo, Sueli G. Rodrigues, Pedro F. C. Vasconcelos, Marcio R. T. Nunes, Richard M. Elliott

**Affiliations:** ^1^​ MRC-University of Glasgow Centre for Virus Research, 464 Bearsden Road, Glasgow G61 1QH, Scotland, UK; ^2^​ Biomedical Sciences Research Complex, School of Biology, University of St Andrews, St Andrews KY16 9ST, Scotland, UK; ^3^​ Department of Cell and Molecular Biology, University of Sao Paulo School of Medicine, 3900, Av Bandeirantes, Ribeirão Preto, SP 14049-900, Brazil; ^4^​ Center for Technological Innovation, Instituto Evandro Chagas, Ananindeua, Brazil; ^5^​ Department of Arbovirology and Hemorrhagic Fevers, Instituto Evandro Chagas, Ananindeua, Brazil

## Abstract

Oropouche virus (OROV) is a public health threat in South America, and in particular in northern Brazil, causing frequent outbreaks of febrile illness. Using a combination of deep sequencing and Sanger sequencing approaches, we determined the complete genome sequences of eight clinical isolates that were obtained from patient sera during an Oropouche fever outbreak in Amapa state, northern Brazil, in 2009. We also report the complete genome sequences of two OROV reassortants isolatd from two marmosets in Minas Gerais state, south-east Brazil, in 2012 that contained a novel M genome segment. Interestingly, all 10 isolates possessed a 947 nt S segment that lacked 11 residues in the S-segment 3′ UTR compared with the recently redetermined Brazilian prototype OROV strain BeAn19991. OROV maybe circulating more widely in Brazil and in the non-human primate population than previously appreciated, and the identification of yet another reassortant highlights the importance of bunyavirus surveillance in South America.

## Introduction

Oropouche virus (OROV) is a midge-borne orthobunyavirus that causes a febrile illness in humans throughout northern South America. The virus is endemic to Brazil and to date all major outbreaks have been limited to the northern region of the country. The largest known OROV outbreak was recorded in 1980 in the state of Para with an estimated 100 000 cases (Anderson *et al.*, 1961; Borborema *et al.*, 1982; Dixon *et al.*, 1981; LeDuc *et al.*, 1981; Pinheiro *et al.*, 1976; Pinheiro, 1962; Vasconcelos *et al.*, 1989). Due to the similarity of signs and symptoms to other endemic viral diseases such as dengue, chikungunya and Mayaro fevers and the lack of a differential surveillance system, the burden of OROV on the Brazilian public health system and economy remains unclear. In an urban environment, the midge *Culicoides paraensis* transmits OROV among humans (Pinheiro *et al.*, 1981, 1982; Roberts *et al.*, 1977), whilst in the tropical forest the virus has been isolated from the pale-throated three-toed sloth (*Bradypus tridactylus*) and the black-tufted marmoset (*Callithrix penicillata*), although the vectors are largely unknown (Nunes *et al.*, 2005a; Pinheiro *et al.*, 1976).

OROV belongs to the genus *Orthobunyavirus*, the largest of the five genera in the family *Bunyaviridae*, which contains several other important human and veterinary pathogens such as La Crosse, Akabane, Cache Valley and Schmallenberg viruses (Elliott, 2014). OROV is classified in the Simbu serogroup and, like all bunyaviruses, contains a tripartite negative-sense RNA genome. The large (L) segment encodes the viral polymerase, the medium (M) segment encodes the viral glycoproteins Gn and Gc, and a nonstructural protein, NSm, and the small (S) segment codes for the viral nucleocapsid protein (N) and a second non-structural protein (NSs) from overlapping ORFs (Elliott, 2014; Elliott & Blakqori, 2011). Recently, we reported the complete genome sequence for the prototype Brazilian OROV strain BeAn19991 (GenBank accession numbers KP052850–KP052852). Our analysis corrected several errors in the previously published OROV genome sequences, most notably that the S segment was 958 nt and not the originally published 754 nt (Acrani *et al.*, 2014).

Here, we report the complete genome sequences of eight clinical isolates of OROV and two primate-derived OROV reassortants. The M segment of the reassortant virus was a unique Simbu sequence that fell in the same clade as the Jatobal virus (JATV) M segment. All 10 isolates contained S segments that were 11 nt shorter than the BeAn19991 strain. To our knowledge, this is the first report of complete genome sequences for OROV field isolates, and we discuss the importance of this in terms of understanding the evolutionary history of the virus.

## Results

### Complete genome sequence of OROV clinical isolates

**Fig. 1.**
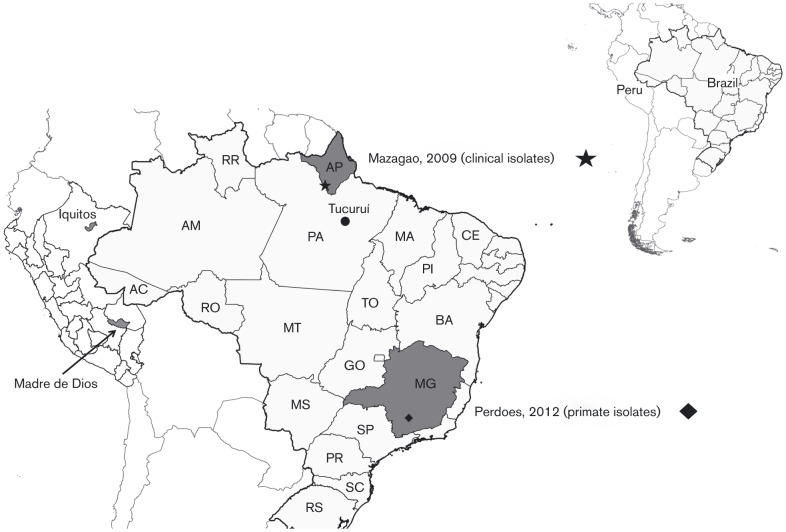
Location of samples sequenced in this study. The map also shows Iquitos and Madre de Dios in Peru where OROV M segment reassortants were isolated, and Tucuruí, a municipality in Para, Brazil, where JATV was isolated. AC, Acre; AM, Amazonas; AP, Amapa; BA, Bahia; CE, Ceara; GO, Goias; MA, Maranhao; MG, Minas Gerais; MS, Mato Grosso do Sul; MT, Mato Grosso; PA, Para; PI, Piaui; PR, Parana; RO, Rondonia; RR, Roraima; SC, Santa Catarina; SP, Sao Paulo; RS, Rio Grande do Sul; TO, Tocantins.

OROV isolates BeH759021, BeH759022, BeH759024, BeH759025, BeH759040, BeH759146, BeH759529 and BeH759620 represent a small portion of OROV samples that were obtained from febrile humans between June and August 2009 in the town of Mazagão, Amapa state, Brazil (Table 1, Fig. 1). The mean age of the patients was 26.5 years and all had presented a similar clinical picture characterized by fever, headache, arthralgia, myalgia and ocular pain. Genome sequences for these isolates were generated by *de novo* assembly of 1 058 075 trimmed and filtered sequence reads obtained using a Roche 454 sequencer.

**Table 1.** Information about samples sequenced in this study

Sample	ID	Isolation date	Host	Country	State	Town	Age (years)	Gender	Source	GenBank accession nos
BeH759021	AMA 2076	23/07/2009	Human	Brazil	Amapa	Mazagao	18	M	Serum	KP691606–KP691608
BeH759022	AMA 2077	24/07/2009	Human	Brazil	Amapa	Mazagao	39	M	Serum	KP691609–KP691611
BeH759024	AMA 2079	24/07/2009	Human	Brazil	Amapa	Mazagao	24	M	Serum	KP691603–KP691605
BeH759025	AMA 2080	24/07/2009	Human	Brazil	Amapa	Mazagao	23	F	Serum	KP691612–KP691614
BeH759040	AMA 2095	23/07/2009	Human	Brazil	Amapa	Mazagao	48	M	Serum	KP691615–KP691617
BeH759146	AMA 2337	20/08/2009	Human	Brazil	Amapa	Mazagao	31	M	Serum	KP691630–KP691632
BeH759529	AMA 2238	17/06/2009	Human	Brazil	Amapa	Mazagao	13	F	Serum	KP691618–KP691620
BeH759620	AMA 2329	23/06/2009	Human	Brazil	Amapa	Mazagao	16	M	Serum	KP691621–KP691623
BeAn789726	PR 4837	2012	*Callitrhix penicillata*	Brazil	Minas Gerais	Perdoes	na	na	Viscera	KP691624–KP691626
BeAn790177	PR 4843	2012	*Callitrhix penicillata*	Brazil	Minas Gerais	Perdoes	na	na	Viscera	KP691627–KP691629

M, male; F, female; na, not applicable.

**Fig. 2.**
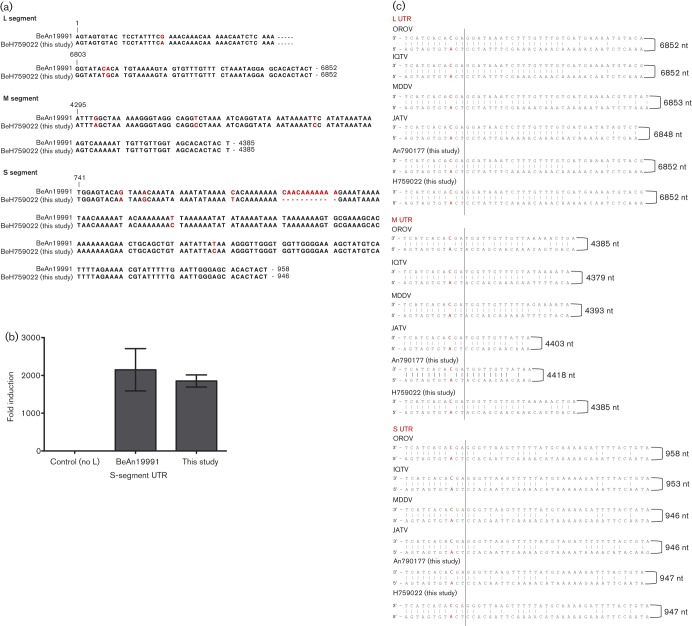
Comparison of UTR sequences. (a) Comparison of the UTRs of BeH759022 isolate (chosen as a representative of the clinical isolates) with OROV strain BeAn19991. The bases in red highlight differences between BeAn19991 and BeH759022. (b) Minigenome assay. Comparison of S-segment-based minigenomes containing the S UTR of OROV BeAn19991 or the S UTR of the newly sequenced isolates. BSR-T7/5 cells were transfected with pTM1OROV-L and pTM1OROV-N plasmids expressing the L and N proteins, respectively, in addition to an S-segment-minigenome-expressing plasmid and pTM1-FF-Luc expressing firefly luciferase as an internal control. The control cells lacked pTM1OROV-L. Minigenome activity was expressed as fold induction over the background control. (c) Comparison of the M-segment UTRs of the novel M segment (BeAn790177) with those of OROV, Iquitos virus (IQTV), Madre de Dios virus (MDDV) and JATV. The C/A mismatch is highlighted in red. The dotted line indicates the extent of the conserved terminal sequence.

The mean S-segment contig length was 867 bases, and by mapping the sequence reads to reference strain BeAn19991 S segment (GenBank accession no. KP052852), we obtained complete S-segment sequences of 947 bases. All S segments were therefore 11 nt shorter than that of the redetermined BeAn19991 strain (Acrani *et al.*, 2014). Ligation of extracted RNA (see Methods) followed by Sanger sequencing was used to confirm the UTR sequences. This revealed that all these isolates lacked nt 781–791 in the S segment of BeAn19991. Additional differences were observed at positions G750A, A754G, C771T, T820C and T888C, resulting in 92.6 % 3′ UTR similarity with BeAn19991 (Fig. 2a). However, despite these differences, promoter activity was similar to that of BeAn19991 (Fig. 2b) when tested in a minigenome assay (Acrani *et al.*, 2014). At the nucleotide level, the N-coding region of these isolates was 95 % similar to that of BeAn19991, but there was 100 % conservation of the translated protein sequence. Unlike in BeAn19991, the NSs-coding region contains tandem AUG translational start codons (a feature of many other orthobunyaviruses; Dunn *et al.*, 1994), caused by C/U variation at nt 56. The NSs ORFs of the human isolates also had a difference at position 332 (A→G), resulting in a Gln→Arg change in the NSs protein at position 89.

The amino acid sequences of the M- and L-segment-encoded proteins of the human isolates were 98.5 and 98.0 % similar to the M- and L-segment proteins of BeAn19991, respectively. We were unable to obtain the terminal sequences of the M and L segments from the deep sequencing data, and therefore 3′ rapid amplification of cDNA ends (RACE) analysis was used. The clinical isolates displayed 99 % similarity among each other across the complete L and M segments, but all had identical UTR sequences that showed 90 and 96 % similarity to the L- and M-segment UTRs, respectively, of BeAn19991 (Fig. 2c).

### Complete sequence of a novel Simbu virus M segment

The sequences of BeAn789726 and BeAn790177, isolates from two black-tufted marmosets (*Callithrix penicillata*), were obtained using deep sequencing and 3′ RACE analysis. The L and S segments showed 99 and 100 % similarity, respectively, to those of the eight clinical isolates. Unexpectedly, the M segment showed only about 56 % similarity at the nucleotide level to other OROV M-segment sequences (about 48 % at the amino acid level). There was, however, a higher similarity with JATV M segment, strain BeAn423380 (GenBank accession no. AFI24667; 71.6 % at the nucleotide level and 76.5 % at the amino acid level). Alignment of the UTR sequences in Fig. 2(c) shows the 11 nt terminal consensus sequence with the conserved C/A mismatch at position 9/−9. This novel M segment was 4418 nt and encoded a 1417 aa polyprotein. Between BeAn790177 and BeAn789726, we observed two nucleotide differences, a silent mutation at position 1676 (U in An790177, C in An789726), and a second at position 1856 (G in An790177, U in An789726) that caused an amino acid change in the translated protein sequence of K or N at position 611 in the polyprotein.

### Phylogenetic analysis

**Fig. 3.**
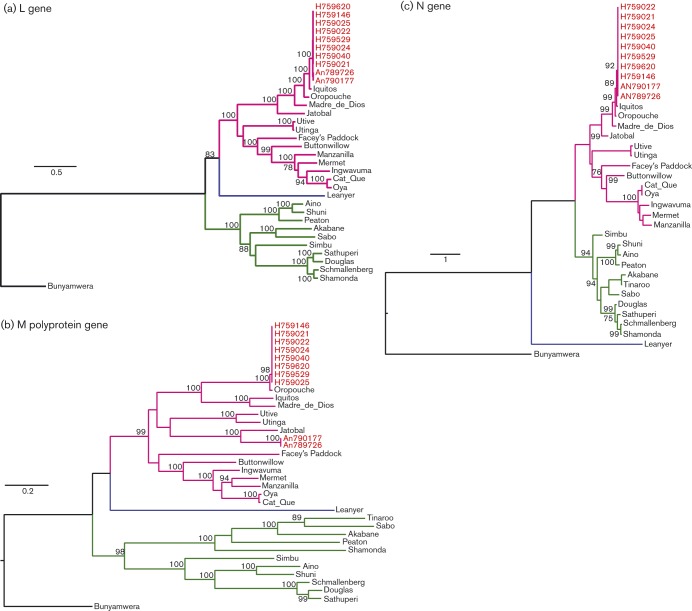
Phylogenetic trees of the Simbu serogroup viruses. The trees were recreated using a maximum-likelihood method based on the general time reversible model (GTR) with five rate categories and assuming sites are evolutionary invariable, for the L gene (a), the GTR model with discrete gamma distribution for the M polyprotein gene (b) and the Tamura three-parameter model with discrete gamma distribution for the N gene (c). Bars, number of nucleotide substitutions per site. Positions with lower than 95 % site coverage were eliminated. Alignment and analysis were conducted in mega6 (Tamura *et al.*, 2013) and final trees were created using FigTree v.1.4.2.

**Fig. 4.**
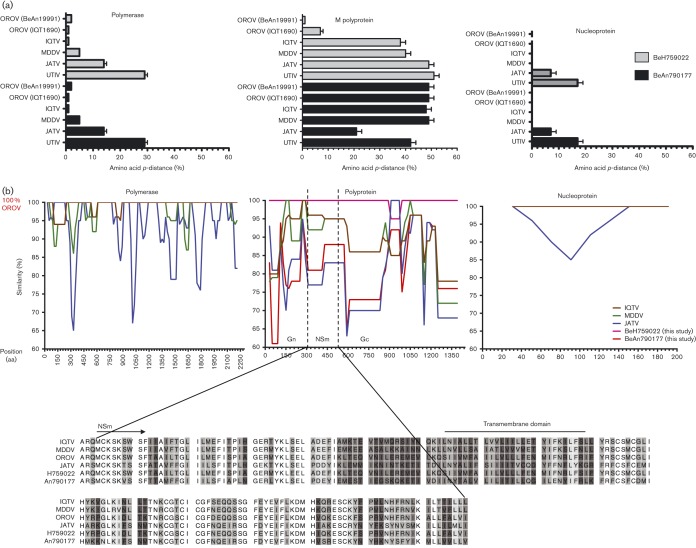
Amino acid comparisons among viruses comprising the species *Oropouche virus*. (a) Pairwise amino acid *p*-distance scores of BeH759022 and BeAn790177 with *Oropouche virus* species and Utinga virus (UTIV). (b) M-segment deduced amino acid similarity plot using OROV as a query sequence and IQTV, MDDV, JATV and BeAn790177 as reference sequences.

To determine the phylogenetic relationship of the newly sequenced isolates within the Simbu serogroup, we compared all available Simbu serogroup virus sequences of the three structural genes, L, M polyprotein and N (Table S1, available in the online Supplementary Material). The eight Amapa state clinical isolates cluster as OROV strains for all L, M polyprotein and N genes (Fig. 3). Pairwise comparisons of the polymerase amino acid sequence for all 10 isolates revealed a pairwise *p*-distance of 2 % towards BeAn19991, but the closest relationship was with Iquitos virus (IQTV) L protein (Fig. 4a). The glycoprotein precursor of the eight clinical isolates had a pairwise *p*-distance value of 1 % towards BeAn19991 (Fig. 4a); however, with samples BeAn790177 and BeAn789726, the glycoprotein gene clustered in a clade close to JATV (Fig. 3b) with an amino acid pairwise *p*-distance value of 21 % compared with 48–49 % with IQTV, OROV and Madre de Dios virus (MDDV) (Fig. 4a). A pairwise sliding-window analysis (Fig. 4b) of BeAn790177, IQTV (strain IQT9924), MDDV (strain FMD1303) and JATV (strain BeAn423380) was performed to analyse the level of similarity in the M polyprotein in comparison with OROV (strain BeAn19991). The highest level of similarity between OROV and BeAn790177 occurred between amino acid positions 1141 and 1341.

### Genetic relationships among members of the species *Oropouche virus*

**Fig. 5.**
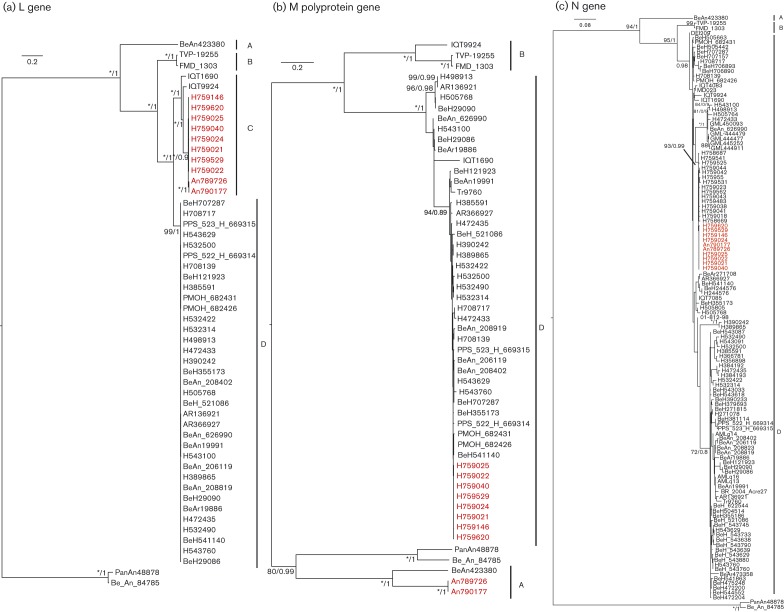
Phylogenetic trees of viruses comprising members of the species *Oropouche virus*. (a) Maximum-likelihood phylogeny of the L gene with bootstrap support/Bayesian posterior probability shown on the branch. (b) Maximum-likelihood phylogeny of the M polyprotein gene with bootstrap support/Bayesian posterior probability shown on the branch. (c) Maximum-likelihood phylogeny of the N gene with bootstrap supports/Bayesian posterior probability shown on the branch. In (a)–(c), * represents 100 % bootstrap support. Isolates sequenced in this paper are highlighted in red. Full details of the strains used in this analysis are presented in Table S2. Bars, number of nucleotide substitutions per site. Clades A–D are indicated.

**Fig. 6.**
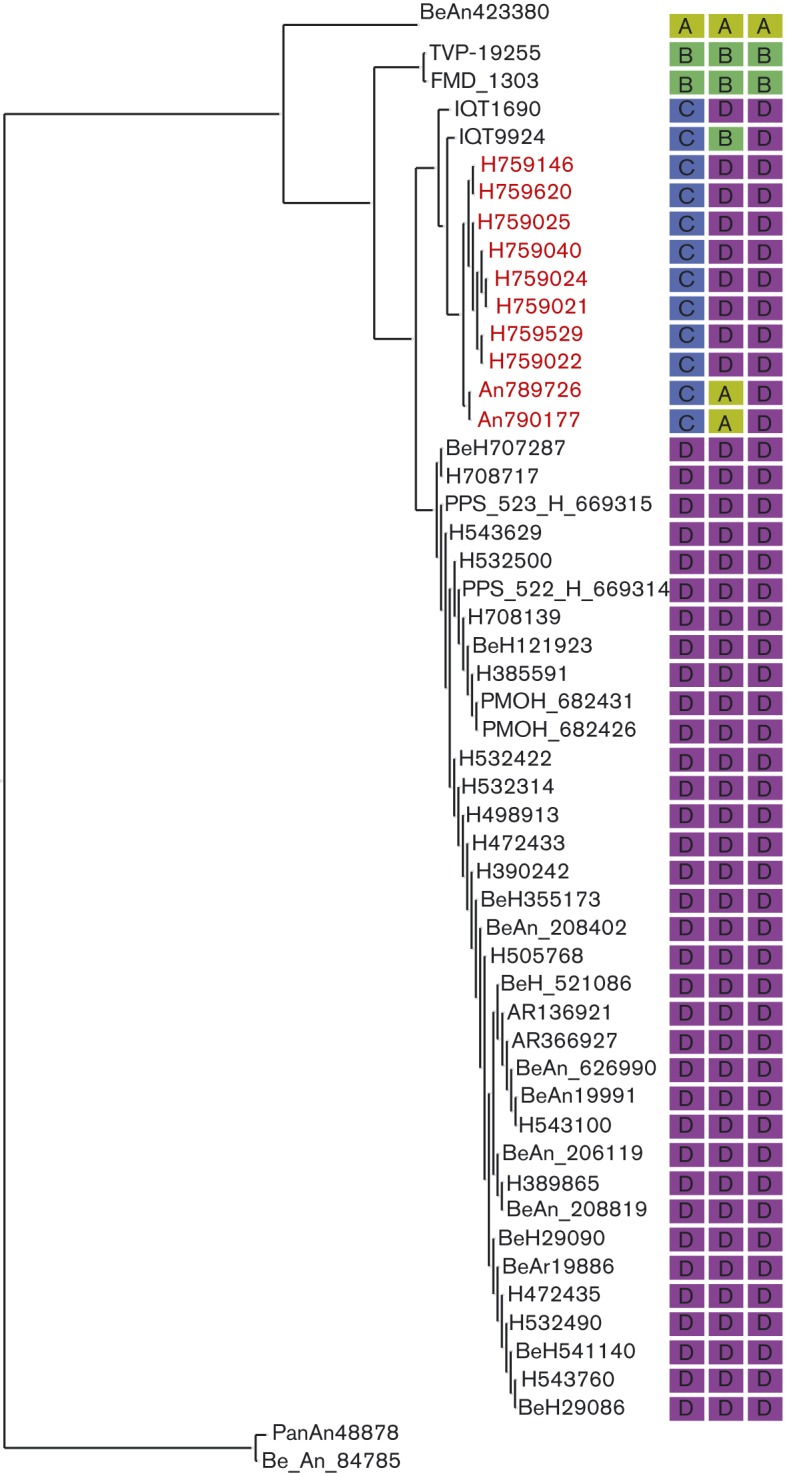
Reassortment among viruses comprising the species *Oropouche virus*. Maximum-likelihood phylogeny of the L segment with each isolate annotated with their clade assignment (A–D) according to the L-, M- and S-segment phylogenies. The different patterns represent the different interclade reassortments: pattern 1, C-D-D; pattern 2, C-B-D; pattern 3, C-A-D; pattern 4, D. Isolates sequenced in this paper are highlighted in red.

OROV showed two clearly identifiable clades for the L and M genes supported by high bootstrap and posterior probabilities (Fig. 5a, b). The trees were topologically different, especially with respect to the M gene of isolates BeAn790177 and BeAn789726, which clustered with high support with JATV (BeAn423380) (Fig. 5b). Interestingly, the Amapa clinical isolates in the L-gene tree clustered with IQTV (IQT9924) and the Peruvian OROV isolate (IQT1690) with high bootstrap support and posterior probability (100 and 1, respectively) (Fig. 5a). The N-gene phylogeny on the other hand was less resolved, with most isolates belonging to a single clade and all being closely related (Fig. 5c). Using a dataset of concatenated genes for each isolate, analysis with the Recombination Detection Program (rdp) recognized four reassortment events with breakpoints close to the gene boundaries, with 33 isolates identified as reassortants (Table 2). Three of these reassortment events were well supported by the gene phylogenies and formed three different mosaic patterns: (i) IQT1690, BeH759021, BeH759022, BeH759024, BeH759025, BeH759040, BeH759146, BeH759529 and BeH759620; (ii) IQT9924; and (iii) BeAn790177 and BeAn789726. These isolates represented inter-clade reassortants (Fig. 6). The fourth reassortment event (Table 2) suggested an intra-clade (D) reassortment, for which there was less phylogenetic support.

**Table 2.** Summary of rdp analysis to determine potential reassortant isolates

Reassortment event number	Breakpoint positions				Reassortment sequence(s)	Minor parental sequence(s)	Major parental sequence(s)	Detection method						
In alignment		In reassortment sequence	
Begin	End	Begin	End	rdp	geneconv	Bootscan	Maxchi	Chimaera	SiSscan	3Seq
1	1	7481	1	7452	BeAn790177, BeAn789726	H759620, TVP-19255, H759040 FMD_1303, BeH759024, BeAn19991, BeH759146, BeH759021, BeH759025, BeH759529, BeH759022	BeAn423380	ns	5.47E−30	ns	9.21E−91	7.86E−67	3.35E−13	5.74E−27
2	7443	1	7416	1	IQT9924	Unknown (BeAn19991)	BeH759022, BeH759040, BeH759024, BeH759146, BeH759021, BeH759025, BeH759529, BeH759620	4.84E−13	2.40E−13	4.13E−12	2.43E−54	1.09E−44	2.51E−10	8.81E−20
3	7368	52	7341	52	BeH759024, BeH759040, BeH759146, BeH759021, BeH759025, BeH759529, BeH759022, BeH759620	BeAn19991	IQT1690	2.31E−57	1.15E−54	2.79E−58	6.96E−26	7.96E−25	5.79E−43	4.92E−48
4	7469	6833	1361	728	BeH472433, BeH355173, BeAn_208402, PPS_522_H_669314, BeH543760, BeAn19991, BeH498913, BeAR366927, PPS_523_Be H_669315, BeH708139, BeH_521086, BeH543100, BeH472435, BeH543629, BeAn_208819, BeH707287, BeH708717, BeAr19886, BeAn626990, PMO Be H682426, BeAR136921, PMO BeH682431	BeH390242, BeH389865	IQT1690, BeAn789726, IQT9924, BeAn790177	2.18E−08	3.79E−08	9.52E−12	1.98E−12	1.33E−07	3.99E−16	1.56E−11

ns, Not significant.

## Discussion

OROV causes a febrile illness in the South American human population, with more than half a million cases reported in Brazil in the last 59 years, and although the annual OROV incidence in the country is unknown, sporadic cases are constantly being picked up, making it a major public health problem. Recently, we corrected the complete genome of the Brazilian prototypic OROV reference strain BeAn19991 (Acrani *et al.*, 2014), a strain that was isolated originally from a pale-throated three-toed sloth (*Bradypus tridactylus*) in 1960 (Pinheiro *et al.*, 1981). In this study, we have described the complete genomic sequences for 10 field samples isolated more recently in Brazil (eight from humans and two from non-human primates), revealing new phylogenetic information on OROV. Phylogenetic analysis of the N gene of several OROV isolates carried out by Saeed *et al.* (2000), and subsequently by several other groups, classified OROV into four genotypes (Aguilar *et al.*, 2011; Azevedo *et al.*, 2007; Nunes *et al.*, 2005a, b; Vasconcelos *et al.*, 2009, 2011). However, the bootstrap values for this classification into four distinct genotypes did not give strong support, prompting us to reanalyse all available OROV sequences in GenBank, along with our newly sequenced field isolates. Our analysis revealed that the N-gene tree lacks structure and that the previously classified genotypes are not clearly distinguishable. The N gene is more conserved compared with the L and M genes where it is possible to distinguish two clades.

Vasconcelos *et al.* (2011) analysed the genetic evolution and dispersal of OROV in South America using samples from 1961 to 2009, the first study aimed at understanding the molecular epidemiology of this human pathogen. However, the results have to be treated with caution as the authors utilized only partial genetic information from each gene and not complete sequences. In the current analyses, complete sequences were analysed. We observed that the S segment 3′ UTR of the field isolates differed from that of BeAn19991 quite significantly (Fig. 2a; residues 781–791 were missing) in both the human and primate virus samples, which were isolated in different geographical regions and at different times (Table 1). For the M-segment UTRs, we noted that the field isolates differed from BeAn19991 at positions G4299A, T4319C and T4343C, whilst for the L segment the differences were observed at G20A, C6809T and A6810G. These findings highlight the need to consider UTR sequences, in addition to coding sequences, when trying to understand the evolutionary history of a virus. Advances in nucleotide sequencing technology mean that full-genome determination is now feasible on a routine basis. The loss of 11 residues in the S segment is intriguing, although it appeared to have no effect on the UTR function when analysed using our minigenome system (Acrani *et al.*, 2014) (Fig. 2b). Previous work, however, has demonstrated that internal deletions in the S-segment UTRs of Bunyamwera virus (BUNV) do not affect virus viability but do interfere with replication causing growth attenuation in cell culture (Lowen & Elliott, 2005). Similar results have also been shown for the BUNV M- and L-segment UTRs (Mazel-Sanchez & Elliott, 2012). The apparent natural deletion of these 11 residues could be important for virus replication efficiency and virus fitness, both *in vitro* and *in vivo*, and are worth pursuing further.

Another interesting finding was the identification of a novel Simbu serogroup virus M segment, in samples BeAn790177 and BeAn789726, obtained from the primate *Callithrix penicillata*. These viruses were isolated in Minas Gerais state, south-east Brazil, 7 years after OROV was first described in this area (Nunes *et al.*, 2005a). Interestingly, the OROV isolate (BeAn626990, GenBank accession no. AY117135) described by Nunes *et al.* (2005a) was also isolated from *Callithrix penicillata*. The S segment of BeAn626990 had a 92 % pairwise sequence identity to the S segments of BeAn790177 and BeAn789726, and clustered separately in the phylogenetic tree (Fig. 5c). L and M sequence information for sample BeAn626990 is currently unavailable, but this virus was identified as OROV based on complement fixation tests that measure antibody responses against the N protein, similar to the way in which the viruses in this study were initially identified as OROV isolates. The fact that OROV has been detected in the area twice is of concern, as it would suggest that the virus is stably circulating in the marmoset population in a region where currently OROV or other Simbu virus outbreaks have not been reported. For epidemiological and phylogenetic research purposes, sequencing of all three segments is crucial so that reassortants such as this are detected. Genetic reassortment is common among segmented viruses such as bunyaviruses (Briese *et al.*, 2013). IQTV and MDDV, both isolated from febrile patients in Peru in 1999 and 2007, respectively, contain L and S segments highly similar to those of OROV, but with M segments that cluster further away from OROV in a sister clade (Aguilar *et al.*, 2011; Briese *et al.*, 2013; Ladner *et al.*, 2014). The L and S segments of the primate-derived virus in this report revealed a similar level of nucleotide identity to that of OROV and IQTV, whilst the M segment was unique and clustered close to JATV. JATV was originally isolated in 1985 from a ring-tailed coati (*Nasua nasua*) in Para, Brazil (Figueiredo & Da Rosa, 1988). In 2001, the S and M segments of JATV were sequenced, classifying this virus as a potential OROV reassortant based on the fact that its N and NSs proteins encoded by the S segment were highly similar to OROV isolates from Peru and that its M segment was unique (Saeed *et al.*, 2001). Recent deep sequencing on the same JATV virus stock now suggests that the S, M and L segments of JATV are more divergent from OROV than initially thought (Ladner *et al.*, 2014). Based on our genetic analysis of the BeAn790177 and BeAn789726 M segments and the significant distance to OROV, IQTV, MDDV and JATV, we propose naming this isolate Perdões virus, after the municipality in which it was isolated.

In this study, we classified the viruses currently comprising the species *Oropouche virus* into clades A, B and D. IQTV fell into its own clade C for the L gene; however, it clustered in clades B and D for the M and N genes, respectively (Fig. 6). In a recent analysis of the species *Manzanilla* and *Oropouche virus*, Ladner *et al.* (2014) suggested that Manzanilla and Utinga viruses could be thought of as distinct strains of a single virus owing to the level of genetic similarity among current members. The authors suggest that this may not be applicable to the species *Oropouche virus* due to the level of M segment differences (Table S3). However, it is possible that these viruses also represent different strains of the same virus but with a higher degree of M-segment divergence. Unlike the L- and S-segment-encoded proteins that function together in RNA synthesis and hence potentially co-evolve together, the M segment codes for the Gc and Gn envelope glycoproteins that are entry binding proteins as well as being major antigenic targets. Selective pressure to produce viable virus in different host species and in different geographical settings could potentially result in higher levels of variation in the M segment. If this were true, we would assume that the non-structural NSm ORF would remain more conserved, and would expect a higher level of variation in the Gn and Gc proteins. It is also interesting to note that most bunyavirus reassortants tend to contain M segments from as-yet-unknown donors (Briese *et al.*, 2013). Pairwise, sliding-window distance analysis of OROV (BeAn19991) and the possible reassortants IQTV, MDDV, JATV and Perdões virus (BeAn790177) indicated an almost equidistant position between IQTV and MDDV, and between the more distant JATV and BeAn790177, with the lowest similarity scores in the N terminus of Gn protein (positions 1–200, Fig. 4b). The similarity pattern for the NSm and Gc ORFs was constant, maintaining the distance between IQTV/MDDV and JATV/An790177 almost unchanged until residue 950, where a sudden variation of sequence divergence could suggest possible recombination. From residues 950 to 1200, we observed a higher degree of variation within a single viral genome for each virus, with a higher percentage of divergence when compared with the rest of the protein. However, this was the region with the highest degree of similarity among all four viral sequences (except OROV), in contrast to what is observed in the rest of the protein, which could suggest that this particular region is subjected to more selective pressure and prone to a higher degree of conservation. It could also suggest that at some point during evolution they all shared the same sequence with a common ancestor, and the distribution to different geographical regions, such as Brazil (Pará, Amazonas, Acre, Rondônia, Amapá Maranhão, Tocantins, Minas Gerais), Peru and Venezuela, to different hosts (humans, *Bradypus trydactulus*, *Callithrix* sp. and wild birds) and to different invertebrate vectors (*Culicoides paraensis*, *Culex* *quinquefasciatus*, *Coquillettidia venezuelensis* and *Ochlerotatus serratus*) allowed a higher degree of variation through natural selection in the whole M segment, but not in this region, nor in the S and L segments (Baisley *et al.*, 1998; Nunes *et al.*, 2005a; Pinheiro *et al.*, 1982; Vasconcelos *et al.*, 2009). This analysis of the amino acid sequences could suggest that these five viruses are all variants of a single species, contrary to the proposal of Ladner *et al.* (2014) based on the nucleotide sequence. It is interesting that the two viruses closer to OROV (IQTV and MDDV) are human isolates, whilst the ones more distant in this analysis were isolated from animals (JATV and An790177), potentially explaining the different selective pressure and the degree of similarity among these viruses. Whatever the case, OROV, at least for now, is more successful as a human pathogen, and further surveillance of orthobunyaviruses in South America could potentially shed more light on the evolution of the species *Oropouche virus*.

## Methods

### Cells and virus.

Vero-E6 cells were grown in Dulbecco’s modified Eagle’s medium supplemented with 10 % FCS. BSR-T7/5 cells that stably express bacteriophage T7 RNA polymerase (Buchholz *et al.*, 1999) were supplied by K. K. Conzelmann (Max von Pettenkofer-Institute, Munich, Germany) and were grown in Glasgow minimal essential medium supplemented with 10 % tryptose phosphate broth, 10 % FCS and 1 mg G418 ml^−1^. Samples used in this study were obtained from the World Health Organization Reference Centre for Arboviruses at the Department of Arbovirology and Hemorrhagic Fevers, Instituto Evandro Chagas (Ananindeua, Brazil). The eight clinical strains of OROV were obtained originally from human patients in 2009 in the municipality of Mazagao, Amapa state, northern Brazil, and had previously been passaged three times in Vero-E6 cells. Viral isolates PR4843 BeAN790177 and PR4837 BeAN789726 were isolated from liver samples collected from two separate *Callithrix penicillata* found dead in the municipality of Perdões, Minas Gerais state, in 2012. A suspension of monkey viscera prepared with PBS (pH 7.4) and antibiotics (penicillin and streptomycin) was used to inject suckling mice (*Mus musculus*) via the intracranial route. Animals were observed daily and collected immediately when disease was evident. A suspension of mouse brain in PBS was then used to infect Vero-E6 cells and virus was harvested 72 h post-infection. Table 1 and Fig. 1 describe the viral isolates used in the study and the geographical locations.

All experiments with infectious viruses were conducted under Biosafety Level 3 conditions.

### RNA extraction, and genome sequencing and assembling.

Virus was harvested and filtered through a 0.2 µm sterile filter and concentrated using polyethylene glycol 8000. The virus aggregate was resuspended in 500 µl PBS and treated with 25 U µl^−1^ Benzonase (Novagen) for 30 min at 37 °C. RNA was extracted using TRIzol reagent (Invitrogen) according to the manufacturer’s protocol and quantified on a Qubit 2.0 Fluorometer (Invitrogen). The genomes were obtained using the following basic steps: (i) cDNA synthesis using random primers (cDNA Synthesis kit; Roche Life Science); (ii) library preparation (second-strand cDNA synthesis and emulsion PCR); and (iii) nucleotide sequencing using both GS FLX 454 (Roche Life Science) and Ion Torrent (Life Technologies) as described previously (Margulies *et al.*, 2005; Rothberg *et al.*, 2011). The SSF (Standard Flowgram Format) files generated by the GS FLX 454 and Ion Torrent machines containing the sequencing trace data were transferred onto a Linux-based computer for analysis. *De novo* DNA sequence assemblers Newbler v.2.6 (GS Assembler, 454 sequencing, Roche) and Celera were used to assemble reads. Adaptors were first trimmed from generated reads and then assembled to generate contigs. These contigs were then compared against sequences in GenBank by performing a blastx search. Using the top hit generated by blastx as a reference sequence, reads were assembled against this to generate more contigs using GS Reference Mapper Software (Roche). Parameters were left at default. Sequences were evaluated for homopolymers before attempting to fill gaps in the genome by the mapping reference method in CLC Genomics Workbench 6 (CLC bio). Scaffold sequences from a consensus of reads and contigs were generated and evaluated before generating the final genome sequence.

### Sanger sequencing.

Sufficient reads could not be generated to complete the L and M segments of samples BeH759024, BeH759529, BeH759620 and BeH759146, and so the incomplete regions were sequenced via Sanger sequencing. Briefly, reverse transcription-PCR was performed using 10 µl TRIzol-extracted RNA and segment-specific forward or reverse primers, with Moloney murine leukemia virus (M-MLV) reverse transcriptase (Promega). PCR was carried out using KOD Hot Start DNA polymerase (Merck) and the amplified products were purified from agarose gel using a gel extraction kit (Wizard kit; Promega), following the manufacturer’s protocol. Entire M segments were amplified using previously described primers OROVMFg and OROVMRg (Acrani *et al.*, 2014) and products were directly sequenced (Table S4). The L segments were amplified as two separate fragments as described previously for BeAN19991 (Acrani *et al.*, 2014) [primers OROVLFg and Ama3082LR (this study), Ama2930LF (this study) and OROVLRg]. Products were cloned separately into the pGEM-T Easy cloning vector and nucleotide sequences were determined using the T7 F and SP6 primers in the first genome walking reaction (Table S4).

### Sequencing the viral 5′ and 3′ termini.

As described previously (Acrani *et al.*, 2014), total cellular RNA from cells infected with the virus was extracted using TRIzol reagent (Invitrogen) at 48 h post-infection. Both the genomic and anti-genomic 3′ ends were obtained by RACE analysis. RNA was polyadenylated (Ambion) for 1 h at 37 °C and then purified using an RNeasy mini kit (Qiagen). Twelve microlitres of this polyadenylated RNA was then used in a reverse transcription reaction with M-MLV reverse transcriptase (Promega) and Oligo d(T)-Anchor primer (Table S4), followed by a PCR using a PCR Anchor primer and a segment-specific primer (Table S4) with KOD Hot Start DNA polymerase (Merck). Amplified products were gel extracted and purified using a gel extraction kit (Promega Wizard kit), followed by Sanger sequencing.

RNA ligation was carried out by denaturing the RNA at 90 °C for 3 min, and the 3′ and 5′ ends were then ligated using T4 RNA ligase (New England Biolabs) for 2 h at 37 °C. The reaction was heat inactivated at 65 °C and purified using an RNeasy Mini kit (Qiagen). cDNA was synthesized using M-MVL reverse transcriptase (Promega) and primer OROSlig1 (Table S4). PCR was performed using KOD Hot Start DNA polymerase (Merck) and primers OROSlig1 and OROSlig2 (Table S4). The PCR products were gel purified and their nucleotide sequences determined.

### Minigenome assay.

The OROV minigenome assay was performed as described previously (Acrani *et al.*, 2014). In brief, subconfluent monolayers of BSR-T7/5 cells in 24-well plates were transfected with 250 ng expression plasmids pTM1OROV-L and pTM1OROV-N, 125 ng S-segment-based minigenome plasmid and 25 ng pTM1-FF-Luc (Weber *et al.*, 2001). At 24 h post-transfection, *Renilla* and firefly luciferase activities were measured using a Dual-Luciferase Reporter Assay kit (Promega).

### Phylogenetic analysis.

Phylogenetic analysis of the 10 isolate sequences was first conducted with available Simbu serogroup viruses (Table S1). The L-, M- and S-segment coding regions were aligned using the muscle algorithm in mega6.06 (Tamura *et al.*, 2013). A model test was then performed on this alignment, and the best DNA substitution model was used to generate the phylogenetic trees for the L, glycoprotein and N ORFs using a maximum-likelihood method in mega6.06 (Tamura *et al.*, 2013), with 1000 bootstrap replicates. Final trees were recreated using FigTree v.1.4.2. Furthermore, a separate analysis of all 10 isolates along with all OROV isolates was conducted. For this, all OROV sequences were downloaded from GenBank and compiled to include a single sequence for each isolate. Each gene segment was aligned according to the protein alignment using clustal Omega (Sievers *et al.*, 2011) and pal2nal (Suyama *et al.*, 2006). Phylogenetic analyses were reconstructed using the general time reversible (GTR)+GAMMA+I substitution model as selected by the Bayesian Information Criterion (BIC) in jModeltest (Darriba *et al.*, 2012). Maximum-likelihood phylogenies were generated in Phyml (Guindon *et al.*, 2010) using 1000 bootstrap replicates and Bayesian tree reconstruction was carried out using MrBayes (Ronquist & Huelsenbeck, 2003) across four chains for 2 million generations sampling every 100 generations, and stationarity was determined from examination of the log likelihoods and the convergence diagnostics. Trees recovered prior to stationarity being reached were discarded, and Bayesian posterior probabilities of each bipartition, representing the percentage of times each node was recovered, were calculated from a 50 % majority rule consensus of the remaining trees.

### Reassortant and genetic divergence analysis.

To examine reassortment, all genes were concatenated for isolates that had complete genomes and the concatenated alignment was analysed in rdp3 (Martin *et al.*, 2010) using the various built-in recombination analysis methods. Genetic distances were calculated at the amino acid level using a pairwise *p*-distance method with complete deletion in mega6.06 (Tamura *et al.*, 2013). A pairwise sliding-window analysis for the M segment at the amino acid level was performed using SimPlot v.3.5.1 (Lole *et al.*, 1999). Using a 200 bp window, 20 bp step, Kimura (two-parameter) and 1000 bootstrap replications, results were plotted in Prism 6.2.
